# Insight into the Common W-Shaped Uneven Solidification Profile in Slab Casting: From Mechanisms to Targeted Strategies

**DOI:** 10.3390/ma18081867

**Published:** 2025-04-18

**Authors:** Hao Geng, Feifei Yang, Shuaikang Xia, Pu Wang, Jinwen Jin, Jiaquan Zhang

**Affiliations:** 1 School of Metallurgical and Ecological Engineering, University of Science and Technology Beijing, No. 30 Xueyuan Road, Haidian District, Beijing 100083, China; 2 Research Institute of Technology, Shougang Group Co., Ltd., Beijing 100083, China; 3 School of Physics and Materials, Nanchang University, Nanchang 330031, China; 4 Ningbo Iron and Steel Co., Ltd., Ningbo 315807, China

**Keywords:** continuous casting slab, W-shaped solidification, nozzle structure, secondary cooling process

## Abstract

This study elucidates the underlying formation mechanisms and mitigation strategies for the W-shaped solidification profile in slab continuous casting. Through the development of a multiphysics coupling numerical model, integrated with measured nozzle cooling characteristics in the secondary cooling zone, the effect of steel flow patterns in mold and non-uniform cooling conditions in the secondary cooling zone on solidifying shell evolution is systematically studied. A principal finding is that wide-face shell erosion, induced by both the radial expansion jet and the lower recirculation, constitutes the primary determinant of uneven shell thickness. An increase in the immersion depth and inclination angle of the nozzle side-hole exacerbates the non-uniformity of the solidified shell. Non-uniform cooling in the secondary cooling zone further amplifies the shell thickness differences, culminating in characteristic dumbbell-shaped solidified shell geometry. Strategic implementation of localized enhanced cooling on the wide face in the secondary cooling zone demonstrates significant improvement in shell uniformity, with implementation efficacy contingent upon a critical process window (Segments 1–6). These findings establish mechanistic foundations and deliver practical guidance for minimizing centerline segregation through optimized continuous casting parameter configuration.

## 1. Introduction

During slab continuous casting, the uniformity of the solidification end morphology directly affects the internal quality of the cast slab, particularly the formation and distribution of centerline segregation. Traditional research has suggested that centerline segregation in slabs primarily originates from solute-enriched steel flow in the mushy zone caused by bulging, solidification shrinkage, and dendritic bridging [1,2]. Mechanical reduction at the solidification end is often used to suppress the flow of molten steel by strain compensation for the mushy zone [3]. When the relative velocity of the liquid flow in the mushy zone approaches zero, it is generally considered to achieve the optimal reduction rate [4,5,6]. However, recent studies have revealed that uneven solidification along the slab width, manifested as a W-shaped solidification end, significantly exacerbates centerline segregation at the 1/8-width position [7,8,9,10,11]. The root cause lies in the fact that the solidification process at the 1/8-width position typically lags behind that at the central region, leading to greater solidification shrinkage of the mushy zone than that at the central region. However, the soft reduction process predominantly based on the solidification progress of the slab center ignores the inconsistency of the solid fraction in the width direction during the slab solidification process, resulting in the mismatch between the strain compensation under soft reduction and the solidification shrinkage of the residual liquid steel at the 1/8-width position [12,13,14]. The research shows that the effectiveness of soft reduction is weakened in suppressing segregation formation and void healing [15,16,17,18]. Moreover, the segregation is usually difficult to completely eliminate through the subsequent heat treatment and rolling processes, and it adversely affects the uniformity of rolled product properties, leading to centerline laminations or hydrogen-induced cracking [19,20,21,22]. This has become a critical bottleneck restricting the performance stability of high-end steel products.

The formation of the W-shaped solidification profile is closely related to the uniformity of solidified shell growth. In fact, the systematic understanding of the W-shaped solidification end is still insufficient, especially when re-examining the phenomenon of non-uniform solidification from the perspective of the entire continuous casting process of the slab. Existing research predominantly focuses on the influence of cooling uniformity in the secondary cooling zone, attributing the non-uniform solidification front to local heat flux difference caused by uneven spray flux distribution across the width direction. Numerical simulations by many researchers suggest that insufficient cooling intensity at the slab edges in the secondary cooling zone delays solidification at the 1/8-width position, resulting in a crater-shaped liquid core [11,12,13,14,23]. Yao et al. also argued that the secondary cooling nonuniformity is the primary cause of the W-shaped solidification ends, independent of molten steel flow in the mold [11]. However, this view fails to explain the persistent occurrence of pronounced W-shaped solidification ends even after optimizing the secondary cooling uniformity. Notably, the intense impact of the steel jet on the narrow face can trigger local shell remelting [24,25,26,27], indicating that the influence of steel flow in the mold on the uniformity of the initial solidified shell may have been underestimated. Saul verified that the scouring effect of molten steel flow makes the shell thickness obvious inhomogeneity on the slab surfaces [28]. Variations in submerged entry nozzle (SEN) parameters, such as immersion depth and side-hole inclination angle, significantly alter the flow field distribution [29,30,31], leading to divergent shell growth rates between the wide-face edge and the central regions. These differences may be further amplified by subsequent non-uniform cooling in the secondary cooling zone, ultimately forming the W-shaped solidification morphology. Unfortunately, the mechanisms by which the mold flow affects solidification uniformity remain poorly understood.

Traditional soft reduction zones fail to effectively accommodate the non-uniform solidification characteristics along the slab width, and current process measures for controlling solidification uniformity yield suboptimal results. Although the Intentional Bulging Soft Reduction (IBSR) technology compensates for local solidification delays through gap adjustment, its narrow operational window increases the risk of internal cracks. Chen et al. and Zhou et al. improved edge deformation using convex roll reduction to compensate for solidification shrinkage, thereby mitigating the centerline segregation to some extent [15,18]. Many scientists have optimized nozzle arrangements to improve slab solidification uniformity, but these methods often neglect the influence of the molten steel flow and typically require extensive industrial trials to determine suitable nozzle configurations [10,11,14,32]. Therefore, there is an urgent need to develop more universal control methods by addressing the root cause of solidification non-uniformity, namely mold flow and secondary cooling conditions.

Based on the 1750 mm × 237 mm slab continuous caster in a steel plant, the influence of molten steel flow in the mold and cooling uniformity in the secondary cooling zone on the formation of W-shaped solidification ends is systematically investigated. By establishing a three-dimensional flow–heat transfer–solidification model and a two-dimensional heat transfer–solidification model, the dominant role of SEN parameters on the initial shell growth and the amplification effect of uneven secondary cooling spray on the solidification front profile are revealed. Furthermore, a localized intensive cooling strategy is proposed to optimize the soft reduction efficiency by regulating the solidification front profile, offering a novel approach to mitigating slab centerline segregation.

## 2. Mathematical Methodology

### 2.1. Model Description

This study is an extension and in-depth exploration of the authors’ previous work [12], employing a multiphysics coupling model to comprehensively analyze the formation mechanism of the W-shaped solidification end during the slab continuous casting. The complex flow behavior of molten steel in the mold significantly influences the growth of the initial solidified shell. To balance computational reliability and efficiency, the model accounts for the impact of flow on solidification while neglecting molten steel flow effects in the secondary cooling zone, where fluid motion is relatively sluggish and the model size is extensive. Instead, the thermal conductivity in the liquid phase zone is equivalently amplified to approximate flow-enhanced heat transfer [33].

In the three-dimensional multiphysics coupling model, molten steel is treated as a viscous Newtonian fluid. The mushy zone is assumed as a porous medium, where the flow of molten steel obeys Darcy’s Law. A damping source term is introduced into the low Reynolds number turbulence model to account for the influence of solidification on the fluid flow [34,35]. The effects of steel grade, mold powder, and thermal contraction on shell growth are neglected. In the two-dimensional solidification model, the heat transfer along the casting direction is negligible, and the model considers two-dimensional heat transfer in the XY plane only. The primary chemical composition of the studied steel grade is listed in Table 1.

### 2.2. Solution Procedure

To clarify the influence of molten steel flow in the mold and secondary cooling uniformity on the W-shaped solidification end during the slab continuous casting, the computational domain is divided into two sub-models.

(a) 3D flow–heat solidification model

**Figure 1:**
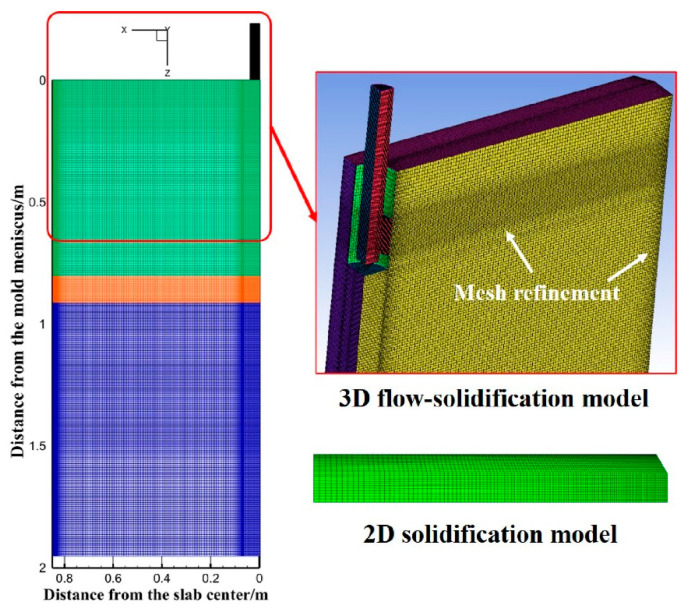
Schematic diagram of model computational domain and meshing.

This model simulates the shell solidification from the mold to Segment 0 of the secondary cooling zone. A quarter model of the slab with a cross-section of 1750 mm × 237 mm is adopted. To ensure fully developed flow in the mold, the effective computational length is set to 4.8 m. The geometric model is illustrated in Figure 1. The coordinate origin is located at the central symmetric point of the meniscus in the mold. The *X*-axis and *Y*-axis correspond to the slab width and thickness directions, respectively, with the positive direction of the *Z*-axis aligned with the casting direction. In this model, the grid is refined where the cooling intensity and flow intensity are large, such as the mold walls and solidifying shell regions. The node spacing at the fine mesh is 2 mm, and the node spacing at the rough mesh is 15 mm. The node space increases at a ratio of 1.1 from the fine grid to the coarse grid. The final mesh for the mold and SEN contains approximately 2.6 million hexahedral elements (Figure 1).

(b) 2D heat transfer–solidification model

This model simulates shell solidification from Segment 0 down to Segment 19 of the secondary cooling zone, covering a distance of 2.0 m to 43.6 m from the meniscus. The temperature field at the outlet of Segment 0 up is extracted in Fluent 2020 R1 and imported as the initial temperature condition for the 2D heat transfer–solidification model.

### 2.3. Boundary Conditions

Considering the insulating effect of mold powder, the molten steel surface is defined as an adiabatic wall with zero shear stress. The inlet of the computational domain adopts a velocity inlet boundary condition. The inlet velocity is determined by mass conservation in continuous casting and calculated from the casting speed, with the temperature set to the pouring temperature. The outlet is assigned a fully developed boundary condition. All the walls are treated as no-slip walls, with zero shear stress at the liquid surface. Heat transfer boundary conditions for the wide and narrow faces of the slab, in both the mold and secondary cooling zone, have been detailed in previous studies [12,35].

To accurately analyze the formation of width-direction non-uniform shell thickness, the actual water flux distribution across the slab width in the secondary cooling zone is measured through a nozzle cold-state performance testing system and applied to the heat transfer coefficient distribution in the 2D model. The testing methodology is well-documented in existing literature [11,36,37].

**Figure 2:**
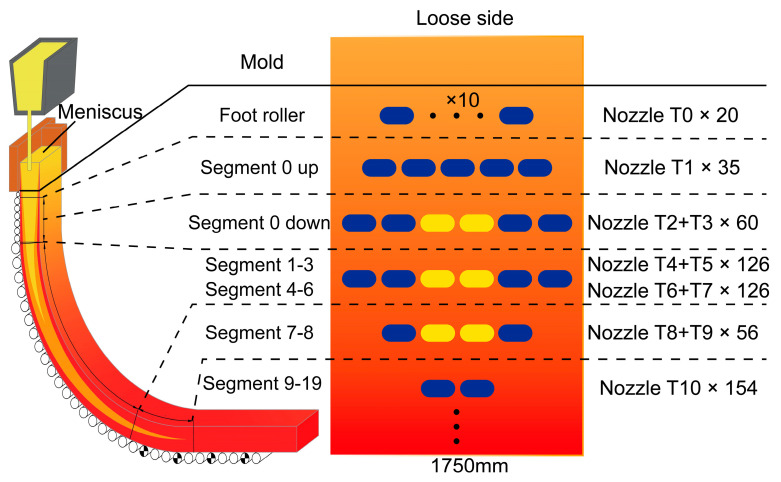
Nozzle type and arrangement in the secondary cooling zone.

The nozzle types and arrangement on the wide face of the secondary cooling zone are illustrated in Figure 2. Except for the foot roller zone, which uses full-water nozzles, all other segments employ air-mist nozzles. The narrow face utilizes full-water nozzles only in the foot roller zone with a water flux of 132 L·min^−1^, while radiation heat transfer dominates in other segments. The foot roller zone is equipped with 10 nozzles of T0 in a row, and Segment 0 up has 5 nozzles of T1. Segments 0 down to Segment 8 have the same nozzle layout, with odd-numbered nozzles on the edges and even-numbered nozzles in the center (specific counts shown in Figure 2). There are 2 nozzles of T10 in a row from Segment 9 to Segment 19. During the testing, the air pressure is maintained at 0.2 MPa (consistent with industrial practice), while water pressure variations are examined to assess their impact on water flux distribution.

**Figure 3:**
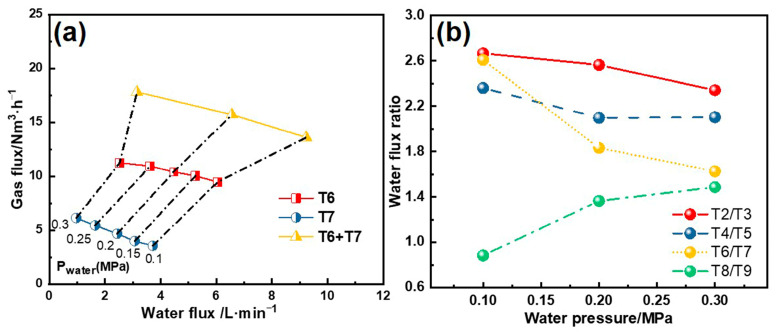
(**a**) Water and gas flux under different water pressures; (**b**) water flux ratio between nozzle types.

Figure 3 shows that as the water pressure increases, the water flow of the nozzles gradually increases, while the gas flow decreases. Additionally, the water and gas flow rates differ for different nozzles. Notably, even-numbered nozzles exhibit significantly higher water flux than the odd-numbered nozzles. However, this ratio gradually decreases with increasing water pressure. For example, in Segments 4–6, the water flux ratio between T6 and T7 nozzles decreases from 2.61 to 1.63, accompanied by reduced differences in peak water flux density. The trend is reversed in Segments 7–8, where the ratio increases, which can be attributed to nozzle structural design. All nozzles exhibit Gaussian-distributed water flux profiles, with primary variations in water flow rate and spray coverage area.

**Figure 4:**
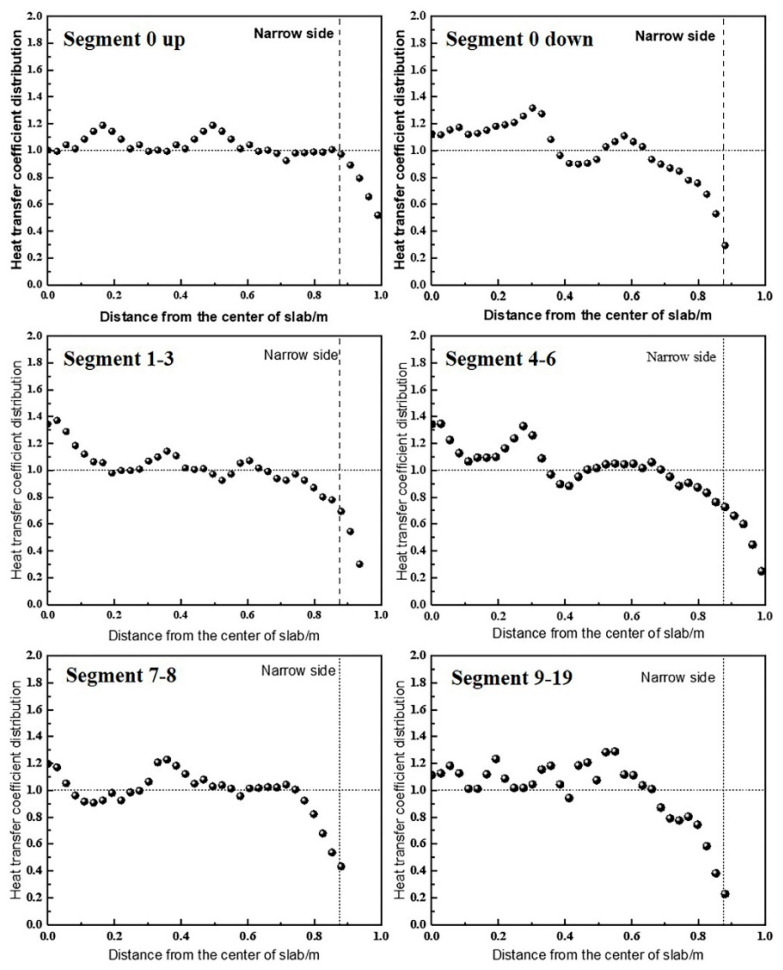
The heat transfer coefficients on the wide surface of each segment.

The distribution of the heat transfer coefficients across the slab width is shown in Figure 4. The foot roller zone, with its limited range and dense nozzle arrangement, is considered to exhibit uniform cooling. In Segment 0 up, the peak heat transfer coefficient appears in the overlapping spray regions of the nozzles, indicating relatively uniform cooling. From Segment 0 down to Segment 8, the water flux density delivered by edge nozzles is lower than that of central nozzles under the same water pressure. In Segments 9–19, two nozzles with larger spray angles (115°) produce uniform heat transfer coefficients without peaks in the central overlap region. The rapid decline in water flux at the edges arises from the Gaussian distribution of individual nozzle sprays, combined with the absence of overlapping sprays in these regions.

**Figure 5:**
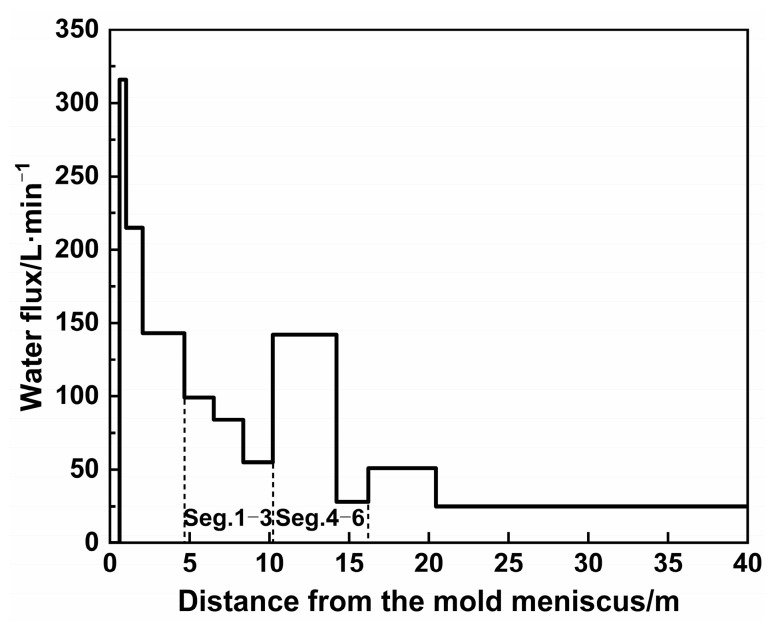
Spray water flux on the wide face in the secondary cooling zone.

The structural and process parameters for the mold numerical simulation are listed in Table 2. The wide and narrow face water fluxes in the mold are 3917 L·min^−1^ and 521 L·min^−1^, respectively, while the secondary cooling water flux distribution is detailed in Figure 5.

### 2.4. Model Validation

**Figure 6:**
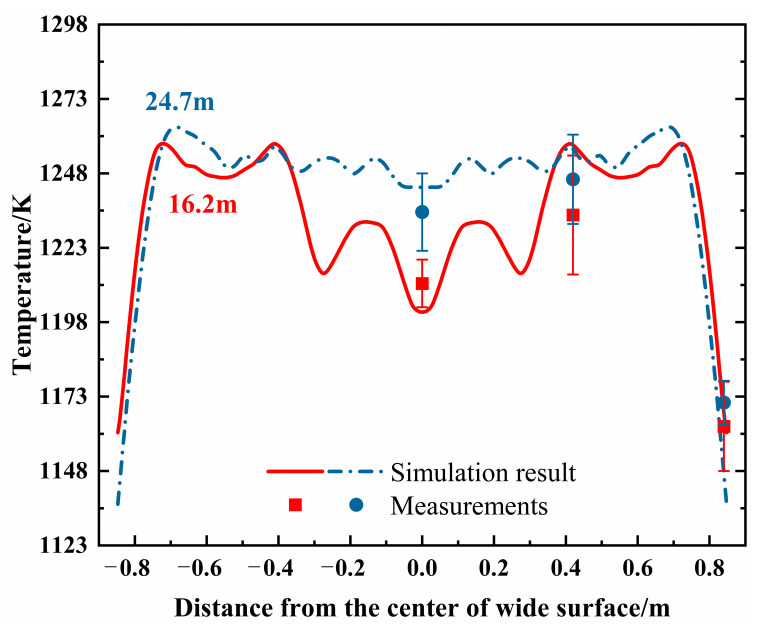
The comparison between the predicted and measured surface temperature.

To validate the model accuracy, the surface temperature profiles across the slab width were measured at 16.2 m and 24.7 m below the meniscus using a calibrated Sciample CIT-G infrared pyrometer. As shown in Figure 6, the predicted temperature at the 1/4-width position is higher than at the center and edges, with more pronounced differences observed at 16.2 m. The maximum relative error between predicted and measured values is less than 4.8%, demonstrating good agreement and confirming the model’s suitability for subsequent analysis.

## 3. Result and Discussion

### 3.1. Mechanism of Formation of W-Shaped Solidification

#### 3.1.1. Initial Solidification Non-Uniformity in the Mold

**Figure 7:**
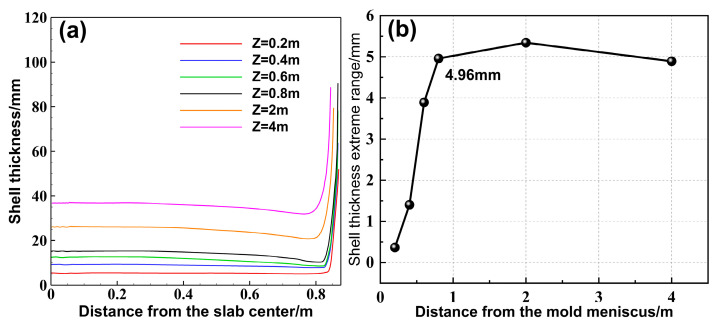
(**a**) Shell thickness, and (**b**) extreme range on wide face of different cross-sections.

The shell growth in the slab caster is closely related to the flow and heat transfer of molten steel. Figure 7 shows the solidification front and the extreme range of shell thickness at different cross-sections within the mold under an immersion depth of 110 mm and an inclination angle of 20°. As the distance from the mold meniscus increases, the shell thickness at the center of the wide face gradually increases, while the shell growth at the wide face corners is slower. Starting from Z = 0.6 m, the shell thickness gradually decreases towards the narrow face, and the extreme range increases sharply, reaching 4.96 mm at the mold exit. Meanwhile, the non-uniform morphology of the shell inside the mold is inherited into the secondary cooling zone, where the extreme range across the wide face first increases and then decreases.

**Figure 8:**
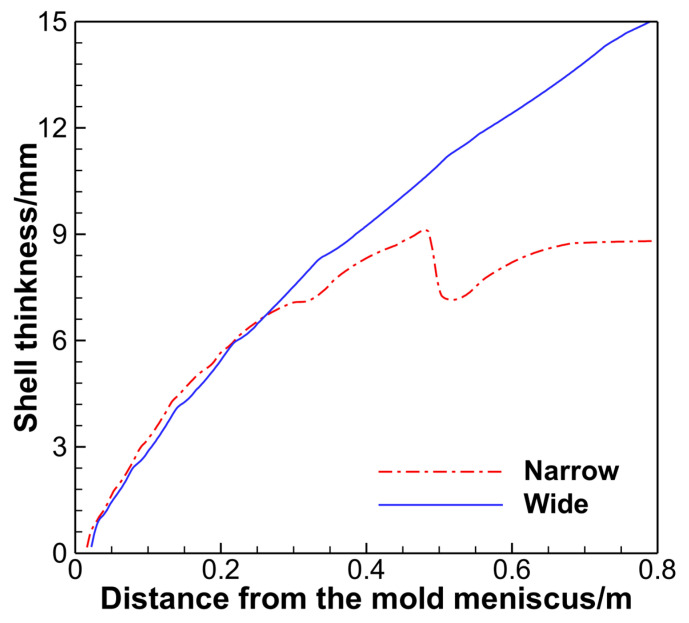
Shell thickness at the center of the wide and narrow surfaces.

Figure 8 presents the shell thickness curves at the centers of the wide and narrow faces. Between 0.3 m and 0.5 m from the meniscus, the growth of the narrow face shell slows down and rapidly thins near 0.5 m, then accelerates again, but the growth rate is slower than that on the wide face. In contrast, the shell growth at the wide face center remains unaffected. This phenomenon occurs because the high-temperature jet from the SEN impacts the narrow face, causing slower growth or even remelting in the impacted zone.

**Figure 9:**
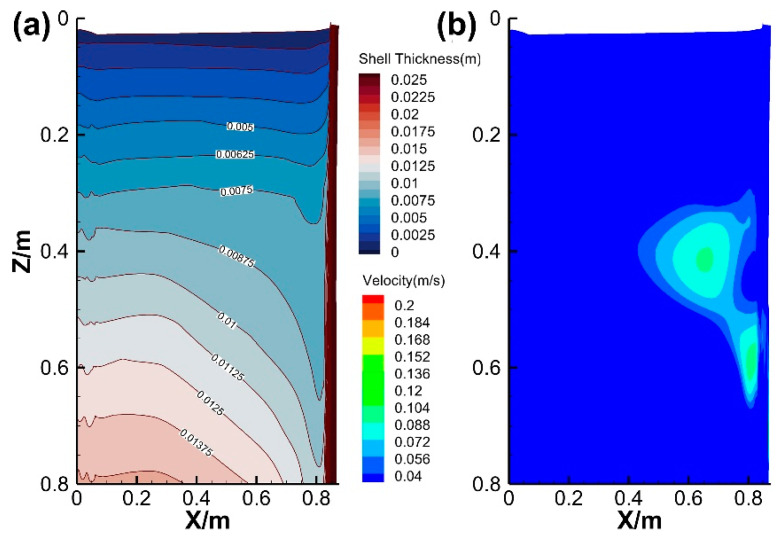
Contours of (**a**) shell thickness and (**b**) flow velocity at the solidification front.

The distribution contours of solidified shell thickness and flow velocity at the solidification front are shown in Figure 9. Within 0.3 m from the meniscus, the shell thickness across the slab width is relatively uniform. However, when the distance from the meniscus exceeds 0.3 m, the shell thickness at the wide face edges significantly decreases, gradually thinning towards the narrow face. As shown in Figure 9b, the highest flow velocity at the solidification front appears at the wide face edges between 0.3 m and 0.7 m from the meniscus, where the shell thickness significantly thins, indicating a close relationship between the decreasing shell thickness gradient and the impact of high-velocity jets.

**Figure 10:**
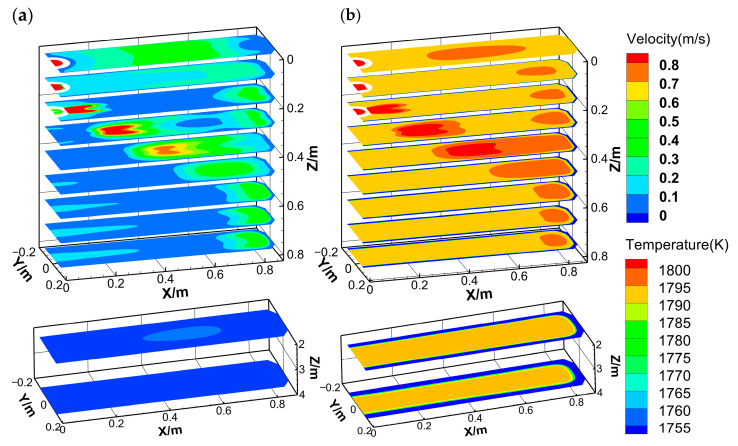
Contours of (**a**) flow velocity and (**b**) temperature at each cross-section.

Figure 10 presents the velocity and temperature fields at different cross-sections. The molten steel jet from the SEN exhibits high velocity and temperature, impinging on the narrow face, subsequently forming a double recirculation flow. A high-temperature zone persists near the narrow face due to two high-speed streams. Besides that, the steel jet contacts the wide face at a distance of 0.4 m from the meniscus due to the radially expanding, while the upper and lower recirculation flows continuously at the solidification front. Combined with the two high-velocity regions at the wide face edge and corner (Figure 9), it is evident that the radial expansion of the steel jet dynamically erodes the wide face edge, causing a decreasing shell thickness in the width direction. The thinnest shell at the corner correlates strongly with the intensity of lower recirculation, while the upper recirculation shows negligible influence on shell growth. After exiting the mold, the flow velocity gradually decreases below 0.03 m·s^−1^. Concurrently, the high-temperature zone near the narrow face gradually disappears, and the molten steel temperature becomes nearly uniform. However, the differences in shell thickness persist, demonstrating that the initial solidification non-uniformity propagates into the secondary cooling zone and ultimately affects the solidification end region.

#### 3.1.2. Effect of Non-Uniform Spray Cooling in the Secondary Cooling Zone

**Figure 11:**
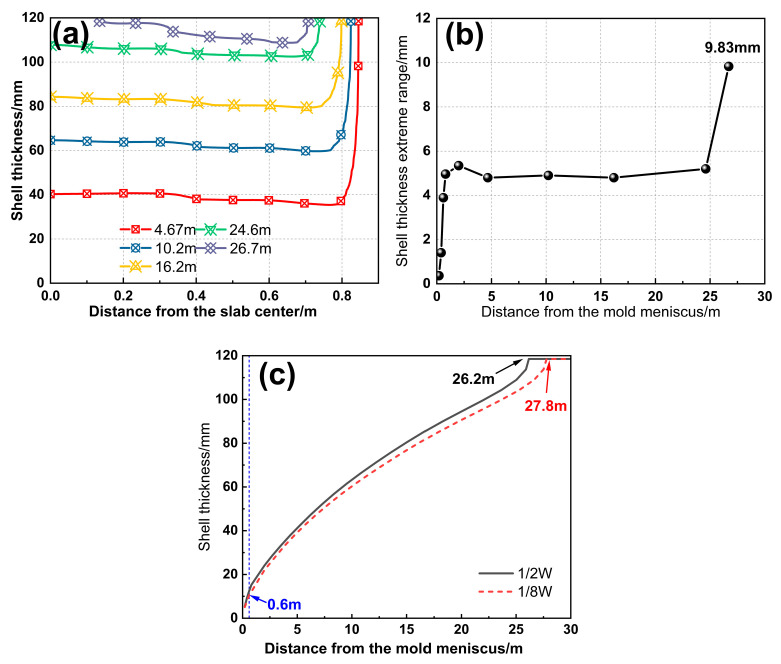
(**a**) Shell thickness and (**b**) extreme range; (**c**) solidification process at the 1/2W and 1/8W positions.

By applying the measured heat transfer coefficient distribution in the width direction (Figure 4) as boundary conditions, the predicted shell uniformity during the actual slab casting reveals a distinct dumbbell-shaped liquid core (Figure 11). After entering the secondary cooling zone, the shell thickness difference slightly decreases at 4.67 m and remains stable for a considerable period. However, at Segment 11 (26.7 m), the slab center completely solidifies, while the 1/8W position retains a thicker liquid core, abruptly increasing the shell thickness difference to 9.8 mm. This occurs because the latent heat release becomes insufficient compared to heat transfer through the solidified shell during the final solidification stage. Besides that, the higher cooling intensity at the center triggers rapid solidification, accelerating shell thickness differences and forming the dumbbell-shaped liquid core. As shown in Figure 11c, the solidification progress at the slab center consistently outpaces the 1/8W position, with a final solidification endpoint discrepancy of 1.6 m, indicating excessive cooling at the wide face center in the secondary cooling zone.

The non-uniformity of slab solidification originates from the steel flow effects in the mold and is amplified by the uneven spray cooling in the secondary cooling zone. Both factors equally contribute to inconsistent shell growth and solidification end. Reassessing the drivers of W-shaped solidification will enable fundamental control of the non-uniform solidification in continuous casting slabs. Additionally, existing 2D solidification models tend to overestimate the solidification non-uniformity. This discrepancy arises because the models neglect the heat transfer along the casting direction, resulting in more pronounced cooling effects at the slab center when spray cooling is applied.

#### 3.1.3. Effect of W-Shaped Solidification on Slab Defects

**Figure 12:**
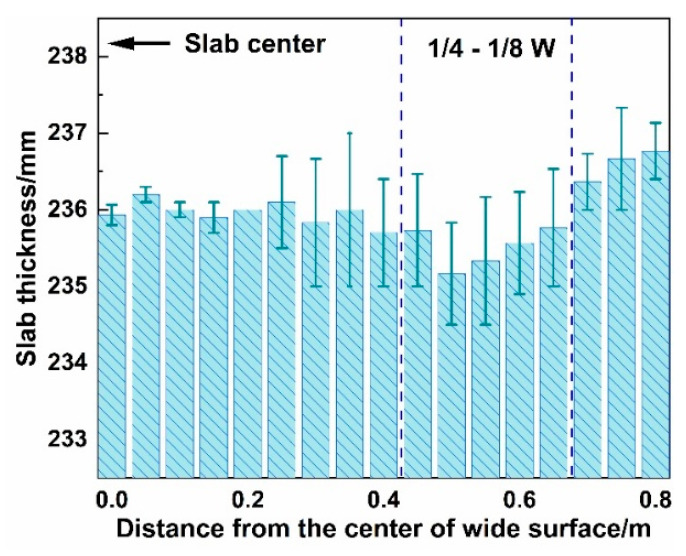
Measurement results of the cold-state slab thickness.

**Figure 13:**
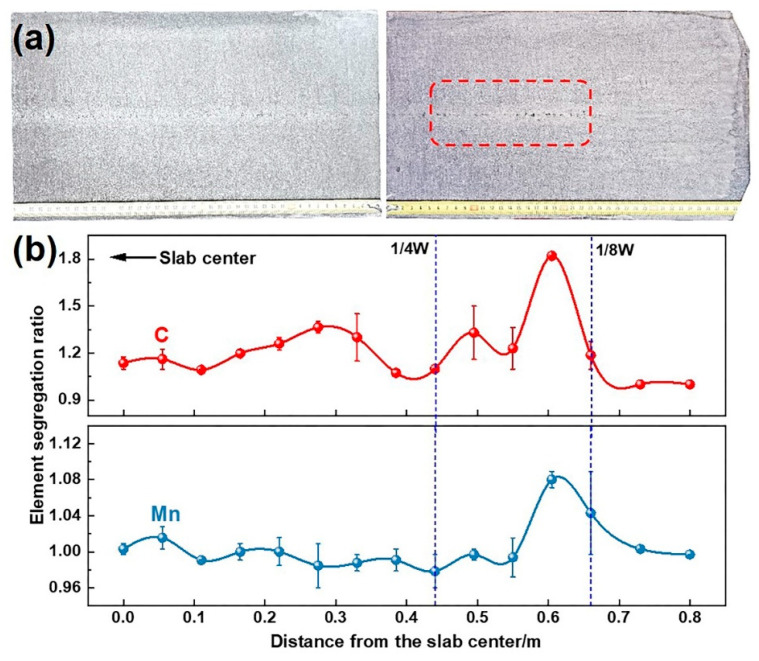
(**a**) Centerline segregation morphology and (**b**) composition distribution of slabs.

Figure 12 shows the measured thickness of the slab at room temperature. The slab thickness in the 1/4–1/8W region is smaller than that at the center and corners, attributed to higher temperatures in this region upon exiting the caster, leading to greater thermal contraction during subsequent cooling. The final centerline segregation morphology of the slab is shown in Figure 13. Dark segregation spots densely populate the 1/8W position along the centerline, with macro-segregation analysis confirming more severe carbon (C) and manganese (Mn) segregation in this region. Combined with Figure 11c, inconsistent solidification endpoints significantly weaken the compensation effect of soft reduction on solidification shrinkage, resulting in severe centerline segregation at the 1/8W positions on both sides of the slab.

### 3.2. Effect of Submerged Entry Nozzle Structure on Solidification Uniformity

**Figure 14:**
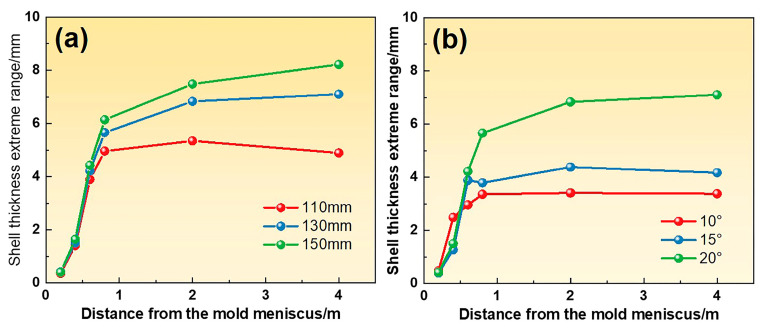
The extreme range of shell thickness for (**a**) different immersion depths with an inclination angle of 20° and (**b**) different inclination angles with an immersion depth of 130 mm.

The structure of the submerged entry nozzle (SEN) critically influences the flow patterns in the mold, ultimately affecting the jet impact depth and shell growth. Thus, optimizing the SEN design is essential. Figure 14 shows the differences in shell thickness with various side hole inclination angles and immersion depths. Numerical simulation results demonstrate that the immersion depth and inclination angle significantly affect the steel flow and shell uniformity in the mold.

As the immersion depth increases from 110 mm to 150 mm, the shell thickness difference at the mold exit increases from 5.0 mm to 6.1 mm, and the shell non-uniformity in the secondary cooling zone is further exacerbated. The increasing side hole inclination angle from 10° to 20° changes the jet angle with a lower impact position on the narrow face and a more intense lower recirculation flow, resulting in a shell thickness difference increasing from 3.3 mm to 5.7 mm. Consequently, the smaller immersion depths and side port inclination angles are beneficial for improving the W-shaped solidification end by enhancing the shell uniformity.

**Figure 15:**
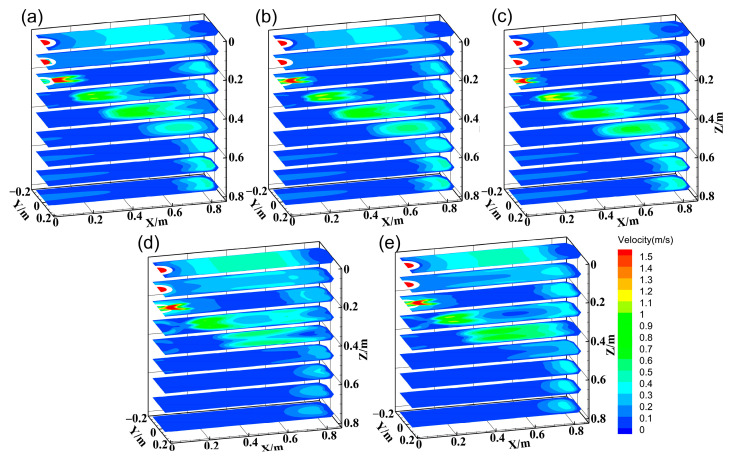
Flow field at different cases. (**a**) 110 mm, 20°; (**b**) 130 mm, 20°; (**c**) 150 mm, 20°; (**d**) 130 mm, 10°; (**e**) 130 mm, 15°.

**Figure 16:**
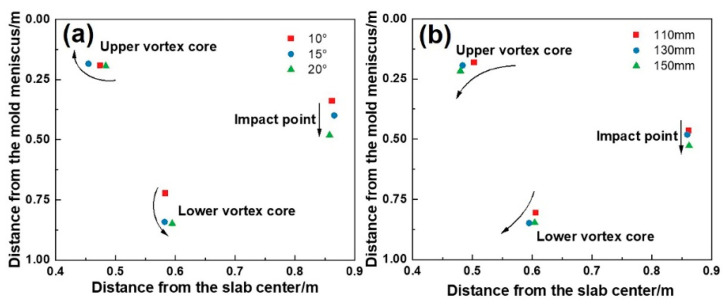
Position of the vortex core and impact point on the narrow face for (**a**) different immersion depths with an inclination angle of 20° and (**b**) different inclination angles with an immersion depth of 130 mm.

These effects arise from altered flow-thermal interactions. Figure 15 illustrates the mechanisms by which the SEN parameters affect flow patterns. The flow field comprises three regions: jet region, upper recirculation, and lower recirculation. As the immersion depth increases, the flow field generally shifts downward, strengthening the lower recirculation while weakening the upper recirculation. Meanwhile, the lower vortex core and the impact position on the narrow face shift downward, as shown in Figure 16, altering the shell erosion regions. High-speed lower recirculation at 0.4–4 m below the meniscus intensifies the erosion of the wide-face corner. However, the shell thickness in regions not directly impacted by the jet gradually increases, further widening the shell thickness difference.

The inclination angle alters the jet direction, thereby regulating the recirculation strength. The steeper inclination angles concentrate kinetic energy in the lower recirculation region [38], increasing the velocity of the lower recirculation significantly. At 10°, the Z-direction velocity component of the jet is relatively low, and due to viscous frictional dissipation, it eventually impacts the narrow face at an upward angle. As the angle increases to 20°, the steel jet impacts the narrow face at a downward angle under the influence of inertial force, exacerbating narrow-face erosion and wide-face corner solidification delays. Meanwhile, the descent of the lower vortex core and impact point is even more significant, reaching 125.6 mm and 140.9 mm, respectively. Both increased immersion depth and inclination angle disrupt the shell growth coordination between the wide face edge and center by redistributing flow energy (weakened upper recirculation and intensified lower recirculation), ultimately degrading solidification uniformity.

### 3.3. Optimization of Solidification Uniformity Based on Localized Enhanced Cooling

Adjusting the submerged entry nozzle (SEN) parameters can mitigate shell nonuniformity in the mold, while the rationality of water flux distribution across the slab width in the secondary cooling zone is equally critical. However, conventional uniform cooling across the wide face often fails to ensure uniform solidification morphology. Therefore, a localized intensive cooling strategy is proposed: enhancing cooling at the 1/4W–1/8W regions (200–400 mm from the slab’s narrow face) while reducing central cooling intensity. This approach aims to compensate for mold-induced shell non-uniformity and avoid exacerbating solidification delay caused by weak corner cooling in the secondary cooling zone. However, implementing intensive cooling in Segment 0 (bending zone) and Segments 7–8 (straightening zone) risks excessive corner temperature reduction and crack initiation. Thus, localized cooling is selectively applied to Segments 1–6 and Segments 9–19.

**Figure 17:**
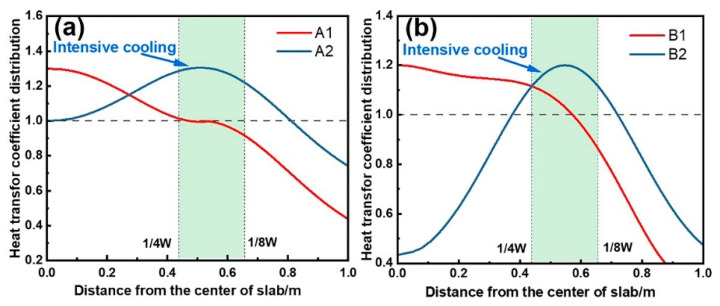
Heat transfer coefficient distribution after equivalent treatment at (**a**) Segments 9–19 and (**b**) Segments 1–6.

To compare the effects of localized intensive cooling across segments, the heat transfer coefficient distributions are equivalently processed while preserving water flux characteristics in the width direction, as shown in Figure 17. A1 and B1 represent the original cooling profiles for Segments 1–6 and Segments 9–19, respectively, with intensive cooling at the center and weak cooling at the edges. Profiles A2 and B2 intensify cooling at the 1/4W–1/8W regions while reducing the central cooling, resulting in a heat transfer coefficient ratio of 1.2–1.3. Four cases are defined in Table 3. Case 1 adopts the original cooling conditions. Cases 2–4 enhance cooling only in Segments 9–19, Segments 4–6, and Segments 1–3, respectively, while maintaining the original cooling conditions in the remaining segments.

**Figure 18:**
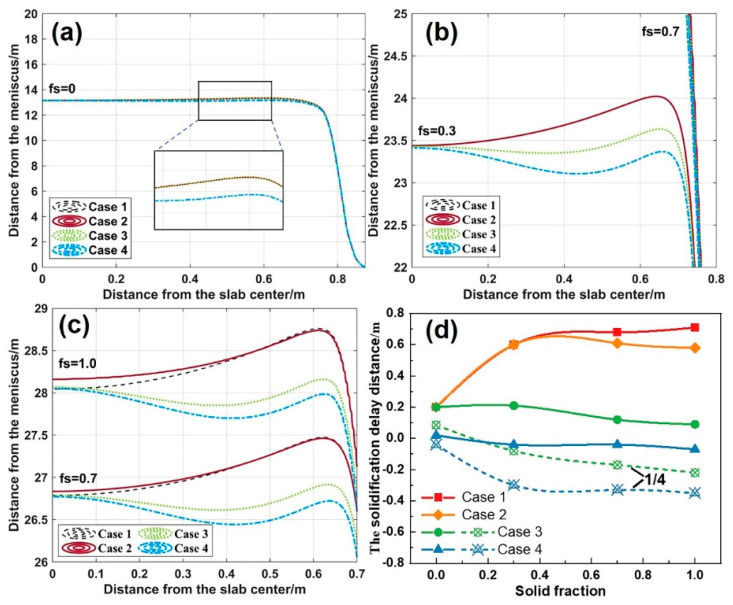
Solid fraction curves at slab centerline: (**a**) fs = 0; (**b**) fs = 0.3; (**c**) fs = 0.7 and 1.0; (**d**) solidification delay distance.

The centerline solid fraction reflects solidification differences across the slab width, critical for developing the soft reduction process. The solidification delay distance is defined as the extreme difference in the distance from the meniscus between a point on the solid fraction curve and the slab center. Figure 18 highlights the distinct solidification delay characteristics along the slab centerline (XOZ plane) across cases. The maximum delay distance reaches 0.71 m in Case 1, while Case 2 reduces the delay distance to 0.58 m. There is a forward-shifted zone of 0.22 m at the 1/4W position in Case 3, and the delayed zone decreases to 0.09 m at the 1/8W position. Moreover, Case 4 exhibits extensive solidification forward shifting at the 1/4W position, with the maximum advancement increasing to 0.35 m.

The evolution of solidification delay distance shown in Figure 18d reveals the differences in the effectiveness of various cooling strategies. In Case 1, the solidification delay distance increases monotonically with the solid fraction (fs). However, the other three cases all show a reduction in the final delay distance, demonstrating improved solidification uniformity in the 1/4W-1/8W regions via the localized intensive cooling in the secondary cooling zone. 

Notably, the impact of localized enhanced cooling on solidification uniformity exhibits significant hysteresis. In Case 2, enhanced cooling (20.4 m below the meniscus) begins to take effect at a distance of 23.4 m from the meniscus. Although the same happens in Case 3 and Case 4, localized enhanced cooling significantly reduces the delay at the 1/8W position. This also suggests that there is a critical operational window for the implementation of localized enhanced cooling (Segments 1–6). Beyond this range, the regulatory efficiency drops sharply.

Additionally, the solidified shell thickness and the water flux are also factors influencing the efficacy of the cooling strategies. Despite the identical heat transfer coefficient ratio of 1.3 in Cases 3–4, Case 4 exhibits more significant solidification forward shifting due to earlier implementation in Segments 1–3. This indicates that the implementation location and the intensity of localized enhanced cooling interact with each other. Appropriately moderating the localized cooling intensity in Segments 1–3 could mitigate the solidification forward shifting and further improve the consistency of the solidification end.

## 4. Conclusions

Through the multiphysics coupling model, this study systematically reveals the formation mechanism and influencing factors of the W-shaped solidification end during the continuous casting slabs. The effects of submerged entry nozzle (SEN) parameters and secondary cooling uniformity on the shell growth and solidification end morphology are investigated. Additionally, an optimized secondary cooling strategy, based on localized intensive cooling, is proposed. The main conclusions are summarized as follows:

(1) The erosion of the shell by the molten steel jet and the lower circulation in the mold leads to initial non-uniform solidification. The non-uniform shell morphology formed in the mold persists into the secondary cooling zone, resulting in the W-shaped solidification end. Increasing the SEN immersion depth and inclination angle promotes lower recirculation development, exacerbating shell non-uniformity within the mold.
(2) The water flux distribution across the slab width exhibits significant non-uniformity, decreasing from the center to the edges. This uneven cooling distribution intensifies the dumbbell-shaped liquid core morphology, with the shell thickness differences sharply increasing near the solidification end. Consequently, the solid fraction at the 1/8-width position is lower than that at other positions, resulting in larger solidification shrinkage of the residual liquid steel and severe centerline segregation at the 1/8-width position.
(3) While the localized enhanced cooling on the wide face effectively promotes the shell growth at the solidification delay region, its influence on solidification uniformity exhibits significant hysteresis effects. A critical optimal operational window (Segments 1–6) is identified for implementing localized enhanced cooling.

## Figures and Tables

**Table 1:** Main chemical components (mass fraction, %).

C	Si	Mn	P	S	Ni	Al	Mo	V	Nb	Ti
0.09	0.010	1.60	0.009	0.002	0.008	0.030	0.002	0.004	0.058	0.014

**Table 2:** Numerical simulation parameter.

Parameter	Value
Slab size/mm^2^	237 × 1750
Casting speed/m min^−1^	1.3
Superheat/K	25
Liquidus temperature/K	1792
Solidus temperature/K	1752
Density/kg m^−3^	7200
Viscosity/Pa s	0.0055
Latent heat/J kg^−1^	2.7 × 10^5^
Thermal conductivity/W m^−1^ K^−1^	29
Thermal expansion coefficient/K^−1^	1.2 × 10^−5^
Specific heat/J kg^−1^ K^−1^	720
Electrical conductivity/S m^−1^	7.14 × 10^5^

**Table 3:** Cooling conditions for each operating condition.

	Segments 1–3	Segments 4–6	Segments 9–19
Case 1	A1	A1	B1
Case 2	A1	A1	B2
Case 3	A1	A2	B1
Case 4	A2	A1	B1

## Data Availability

The original contributions presented in the study are included in the article; further inquiries can be directed to the corresponding authors.
